# Potentially modifiable risk factors for dementia putting the evidence together: Total population attributable fraction

**DOI:** 10.1002/alz.085063

**Published:** 2025-01-09

**Authors:** Gill Livingston, Jonathan D Huntley, Kathy Y Liu, Sergi Costafreda Gonzalez, Geir Selbaek, Suvarna Alladi, David Ames, Sube Banerjee, Alistair Burns, Carol Brayne, Nick C Fox, Cleusa P Ferri, Laura N. Gitlin, Robert J Howard, Helen C Kales, Mika Kivimaki, Eric B Larson, Noeline Nakasujja, Kenneth Rockwood, Quincy M Samus, Kokoro Shirai, Archana Singh‐Manoux, Lon S. S. Schneider, Sebastian Walsh, Yao Yao, Andrew Sommerlad, Naaheed Mukadam

**Affiliations:** ^1^ University College London, London United Kingdom; ^2^ Camden and Islington NHS Foundation Trust, London United Kingdom; ^3^ University of Exeter, Exeter United Kingdom; ^4^ Division of Psychiatry, University College London, London United Kingdom; ^5^ Faculty of Medicine, University of Oslo, Oslo Norway; ^6^ National Institute of Mental Health and Neurosciences [NIMHANS], Bengaluru, Karnataka India; ^7^ The University of Melbourne, Melbourne, VIC Australia; ^8^ University of Nottingham, Nottingham United Kingdom; ^9^ University of Manchester, Manchester United Kingdom; ^10^ Cambridge Public Health, University of Cambridge, Cambridge United Kingdom; ^11^ Universidade Federal de São Paulo (UNIFESP), São Paulo, São Paulo/SP Brazil; ^12^ Drexel University, Philadelphia, PA USA; ^13^ Division of Psychiatry, University College London, London, London United Kingdom; ^14^ University of California, Davis, Sacramento, CA USA; ^15^ University of Washington, Seattle, WA USA; ^16^ College of Health Sciences, Makerere University, Kampala Uganda; ^17^ Dalhousie University, Halifax, NS Canada; ^18^ Johns Hopkins University, Baltimore, MD USA; ^19^ Osaka University, Osaka Japan; ^20^ Université de Paris, Inserm U1153, Epidemiology of Ageing and Neurodegenerative Diseases, Paris France; ^21^ Keck School of Medicine of USC, Los Angeles, CA USA; ^22^ Cambridge Public Health, Cambridge United Kingdom; ^23^ Peking University, Beijing China

## Abstract

**Background:**

Our authors from around the world met to summarise the available knowledge, decide which potentially modifiable risk factors for dementia have compelling evidence and create the most comprehensive analysis to date for potentially modifiable risk factors to inform policy, give individuals the opportunity to control their risks and generate research.

**Method:**

We incorporated all risk factors for which we judged there was strong enough evidence. We used the largest recent worldwide meta‐analyses for risk factor prevalence and relative risk and if not available the best data. We performed new meta‐analyses for depression and hearing loss relative risks.

We used all 37,000 participants aged ≥ 45 years from HUNT, Norwegian longitudinal population‐based study, to estimate communalities (risk factors clustering) as people frequently have multiple risk factors. Four principal components explained 51% of total risk factors variance. We then calculated weighted population attributable fraction (PAF) estimates.

**Result:**

We will present each potentially modifiable dementia risk factor’s prevalence, communality, relative risk, unweighted and weighted PAFs and our new lifecourse infographic.

**Conclusion:**

Our results give hope suggesting many dementias can be prevented or delayed. Many risk factors are linked to deprivation, for example, where people live and exposure to air pollution, or finding affordable healthy food within walking distance and having the resources and skills to prepare it. We have more evidence that longer exposure to a risk has more effect, for example in diabetes, and that risks have more effect in otherwise vulnerable people, for example air pollution. Thus, it is important to redouble efforts to treat existing conditions in communities and people with multiple risks where approaches beyond individual treatment or behaviour change have potentially larger impact. While association is not causation, the effect on cognition of multicomponent, hearing aid and hypertensions RCTs, and naturalistic changes with reduction in air pollution, cigarette smoking, social contact, hearing and vision corrections and work cognitive stimulation, continue to suggest causal relationships with dementia. Socially disadvantaged groups in all countries are more at risk and should be prioritised for intervention. There is more evidence that risks are also modifiable for people at increased genetic risk.

